# A descriptive report on short QT interval in Kherameh branch of the PERSIAN cohort study

**DOI:** 10.1038/s41598-022-06835-y

**Published:** 2022-02-21

**Authors:** Mohammad Hossein Nikoo, Alireza Heiran, Fardin Mashayekh, Abbas Rezaianzadeh, Abbas Shiravani, Fatemeh Azadian

**Affiliations:** 1 Non-communicable Diseases Research Center, Shiraz University of Medical Sciences, Shiraz, Iran; 2 Cardiovascular Research Center, Shiraz University of Medical Sciences, Shiraz, Iran; 3 Student Research Committee, School of Medicine, Shiraz University of Medical Sciences, Shiraz, Iran; 4 Colorectal Research Center, Shiraz University of Medical Sciences, Shiraz, Iran

**Keywords:** Epidemiology, Cardiovascular diseases

## Abstract

Short QT-interval is a condition that bear the suspicion of short QT syndrome (SQTS). SQTS is known to increase risk of life-threatening arrythmias and sudden cardiac death (SCD). Due to the insufficient population-based studies and use of various QT cut-off values, it accounts for as an undiagnosed condition. In this study, we sought for prevalence of short QT interval in Kherameh cohort study, one of the southern branches of the Prospective Epidemiological Research Studies in Iran (PERSIAN). Data of 4363 adult subjects were analyzed from phase 1 of the cohort during 2014–2017. The corrected QT (QTc) intervals were calculated and electrocardiograms (ECGs) with QTc of less than 370 ms (msec) were reanalyzed for bradycardia, early repolarization, atrial fibrillation (AF), arrhythmias, and other electrical conduction abnormalities. Seventy-two subjects (1.65%) had a QTc of less than 370 ms (mean QTc of 360.72 ± 11.72). A male predominance and a lower mean heart rate observed in SQTS susceptible group (M/F of 1/0.26 vs. 1/1.145, *p*-value < 0.0001; 58.389 ± 9.787 vs. 70.899 ± 11.775; *p*-value < 0.0001) compare to the subjects with normal QTc. At least, 2 subjects with high-probability SQTS and 3 with intermediate-probability SQTS identified. The frequency of AF, syncope, bradycardia, early repolarization, low voltage ECG, and infantile SCD in first- and second-degree relatives were 16.67, 4.17, 33.33, 11.11, 11.11, 11.11%, respectively. The prevalence of short QT interval in our cohort was in line with previous studies. The incidence of cardiac symptoms/events, familial SCDs and ECG derived specific findings were high amongst SQTS-susceptible index persons. However, these variables could not predict the symptomatic subjects, which emphasizes gene studies and family screening.

## Introduction

Sudden cardiac death (SCD) amongst individuals without structural heart diseases is a challenging issue, which suspense the non-structural or electrical heart diseases^1^. During late 90s, regardless of definitions of long QT syndrome, Brugada syndrome or familial form of catecholaminergic polymorphic ventricular tachycardia, SCDs were still observed with unknown etiology^2–5^. As it happened, in 2000, Gussak and colleagues^6^ described a novel cardiac channelopathy that was accompanied with familial clustering and SCDs. It was characterized by a constant and idiopathic short QT interval in electrocardiogram (ECG), named short QT syndrome (SQTS).

SQTS in a rare conductive myocardial disorder and it can be hereditary, mostly autosomal dominant (with incomplete penetrance) due to cardiac potassium or calcium channelopathies. It results in accelerated repolarization, decreased refractory period and subsequent increased risk of ventricular arrythmias^7–9^. The most prevalent presentations are palpitation, syncope, atrial fibrillation, SCD, and abnormal ECG in asymptomatic individuals^7,10–12^.

Due to the lack of standard diagnostic criteria, certain QT cut-off value and knowledge about SQTS risk factors, the epidemiological aspects of SQTS are doubtful. However, several cohorts on the normal populations showed that the short QT interval prevalence ranged from 0.37 to 2% at 360 ms cut-off in U.S.^13^ and Japanese^14^ cohorts to 0.02–0.1% at 340 ms cut-off in the Swiss^15^ and Finnish^16^ cohorts, respectively. However, further reports of short QT interval are demanded, which can be potentially obtained through large short QT cohorts^17^. Hence, SQTS accounts for as an undiagnosed condition, because of insufficient literature reports (approximately 300 cases after two decades) and the asymptomatic proportion of cases reflected by the presence of a high risk of malignant arrhythmias and SCDs. In this study, we sought to yield the descriptive aspects of short QT interval in Kherameh cohort study in Fars province, southern Iran.

## Methods and materials

### Study population

We performed a descriptive cohort study on Kherameh cohort, which is one of the southern branches of “Adult Cohort” component of the larger nationwide cohort program, Prospective Epidemiological Research Studies in Iran (PERSIAN) lunched in 2014 (Its objectives, structure and infrastructures is described further^18^). The “Adult Cohort” is the main component of PERSIAN cohort study that was designed to enroll approximately 180,000 40–70-year-old individuals within 18 different cohort sites. All methods were performed in accordance with relevant guidelines and regulations. Kherameh county is located in East-central Fars province, with estimated population of 61,580 people mostly from “Fars” ethnicity. The main objective of Kherameh cohort study was to determine the incidence and risk factors of non-communicable diseases during a follow up period of 10 to 15-years.

According to the PERSIAN cohort whitepaper^18^, Initially, each subject was invited to participate in the study and come to the cohort center. After signing written informed consent, registration and allocating a unique identification number, anthropometric, physical activity, physiological, lifestyle, nutritional, and environmental data were documented and biological samples were collected. When this standard data collection was carried out, three sites, including Kherameh branch, also performed baseline electrocardiography for the subjects. This population was the target of the present study.

The baseline standard 12-lead ECGs (CARDIAX computerized ECG, Medusoft Pty. Ltd., Australia) had been obtained at a paper speed of 25 mm/s and a gain of 10 mm/mV; and machine-derived QTc intervals were recorded. We excluded those who had not baseline ECG (e.g., non-cooperation, aged 35–40 years, etc.), had corrected QT (QTc) interval less than 370 ms but their SQTS survey data were unavailable, were duplicated data, had not standard ECG, or their ECG parameters was not logical (e.g., inaccurate data entry, etc.).

This study was approved by the local Ethics Committee of Shiraz University of Medical Sciences (code: IR.SUMS.MED.REC.1399.544). All the subjects signed the written informed consent.

### Assessments

The upper limit of QTc in a well-studied topic, but the definite cut-off values for SQTS is remained a puzzle nonetheless^19^. It should be noticed that to diagnose SQTS a diagnostic panel might be a better tool rather than single QTc; since, affected and healthy subjects pose a wide overlapping QTc range. In the present study, we applied Gollob criteria^19^ and European Society of Cardiology criteria^20^ for case finding; however, findings were reported based on Gollob criteria (Table 1).

**Table 1:** Gollob and European Society of Cardiology criteria for diagnosing SQTS.

Gollob criteria^18^	1—High-probability SQTS: ≥ 4 points
	2—Intermediate-probability SQTS: 3 points
	3—Low-probability SQTS: ≤ 2 points
	***Item 1 (QTc):*** < 370[1 point], < 350[2 points], < 330[3 points], J_point_-T_peak_ < 120[1 point]
	***Item 2 (Clinical history):*** SCD[2 points], polymorphic VT or VF[2 points], unexplained syncope[1 point], AF[1 point]
	***Item 3 (Familial history):*** 1st or 2nd degree high-probability SQTS[2 points], 1st or 2nd degree autopsy-negative SCD[1 points], sudden infant death syndrome[1 point]
	***Item 4 (Gene study):*** genotype positive[2 points], mutation of undetermined significance in culprit gene[1 points]
European Society of Cardiology criteria^19^	1—QTc < 340 ms, or
	2—QTc < 360 ms and (1) confirmed pathogenic mutation, or (2) family history of SQTS, or (3) family history of SCD at 40 years of age, or (4) Survival from a VT/VF episode in the absence of heart diseases

Within the conduct of this study, we rechecked the QTc interval, manually, through calculating Bazett’s equation (QTc = QT/ ($\sqrt{RR}$). Then, ECGs with QTc of less than 370 ms—labeled as SQTS susceptible group—were reanalyzed by an experienced cardiologist with special qualification in clinical electrophysiology (MHN) from the original paper, and recorded ECG findings i.e., bradycardia, early repolarization, atrial fibrillation (AF), arrhythmias, other electrical conduction abnormalities, etc. Furthermore, demographic variables, past medical history (i.e., cardiovascular diseases, myocardial infarction, chronic headache, epilepsy), history of SCD in subjects and their first- and second-degree relatives, history of sudden infantile death, history of syncope, cardiac diseases in first- and second-degree relatives were recorded. These data were extracted from PERSIAN cohort database or online survey through dedicated platform by local residents who had relevant collage education and underwent practical training through a number of workshops. This online survey included the variables out of the original cohort and performed after permission of the central execution and monitoring committee.

### Statistical analysis

Statistical analysis was carried out using statistical package for social sciences (SPSS) (IBM Corp. Released 2019. IBM SPSS Statistics for Windows, version 26.0. Armonk, NY: IBM Corp.). Qualitative variables and quantitative variables were described using frequency (percent) and mean ± standard deviation (SD) or median [interquartile range (IQR)], respectively. Independent *t*-test and Fisher’s exact test (or Pearson’s Chi square test) were applied to compare QTc > 370 ms group and QTc < 370 ms. *P* ≤ 0.05 was considered statistically significant.

### Ethics approval and consent to participate

This study was approved by the local Ethics Committee of Shiraz University of Medical Sciences (code: IR.SUMS.MED.REC.1399.544). All the subjects signed the written informed consent.

## Results

**Figure 1:**
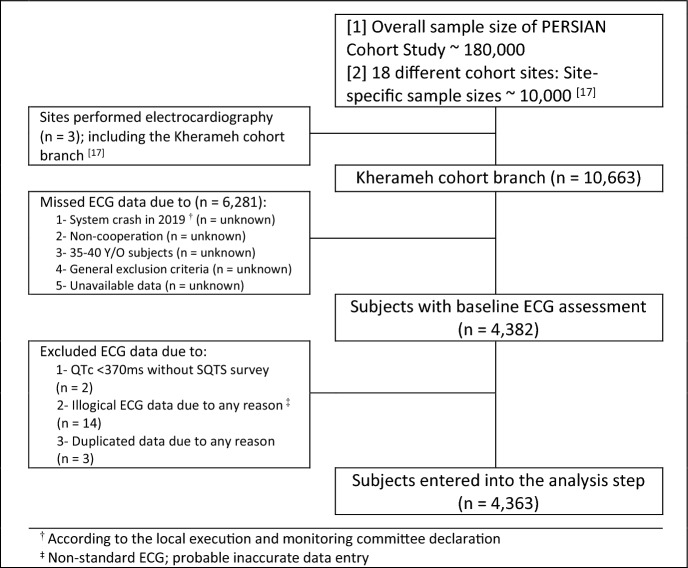
Flowchart of the included subjects from phase I of Kherameh cohort study.

Figure 1 is the flowchart of the included subjects. We analyzed data of 4363 subjects that posed the baseline ECG assessment out of 10,663 subjects aged 40–70 years from phase 1 of the cohort during 2014–2017.

According to Gollob et al.^1^ proposed diagnostic criteria for diagnosing SQTS, 72 subjects (1.65%) were eligible to assess for SQTS with a mean QTc of 360.72 ± 11.72. Compare to the remaining 4291-subject sample with QTc of more than 370 ms, at the baseline, SQTS susceptible group had a significantly higher myocardial infarction events (8.3% vs. 2.3%; *p* = 0.008), but no significant difference for cardiovascular diseases (*p* = 0.100), epilepsy (*p* = 0.425), and chronic headache (*p* = 0.726). We observed statistically significant male predominance in SQTS susceptible group (male/female ratio of 1/0.26 vs. 1/1.145; *p* < 0.0001) but no significant difference for the mean age (51.56 ± 8.41 vs. 52.61 ± 8.08; *p* = 0.788). Moreover, SQTS susceptible group had a significantly lower mean heart rate (58.389 ± 9.787 vs. 70.899 ± 11.775; *p* < 0.0001) (Table 2).

**Table 2:** Comparing baseline demographic, clinical and ECG-derived variables between SQTS susceptible subjects with those with QTc > 370 ms.

	SQTS susceptible (n = 72 (1.65%))	QTc > 370 ms (n = 4291 (98.45%))	*p*
Age, *year* (mean ± SD) [range]	51.56 ± 8.41 [40–72]	52.61 ± 8.08 [40–72]	0.788^‡^
Male/female ratio	1/.26	1/1.145	< 0.0001^†^
Heart rate (mean ± SD; median [IQR]) [range]	58.389 ± 9.787; 58 [7.5] [41–84]	70.899 ± 11.775; 70 [15] [40–134]	< 0.0001^‡^
QTc, ms (mean ± SD; median [IQR]) [range]	360.72 ± 11.72; 365 [10.75] [318–369]	423.24 ± 29.54; 422 [32] [370–768]	< 0.0001^‡^
**Past medical history [frequency (%)]**			
CVDs	11 (15.3)	425 (9.9)	0.100^†^
Myocardial infarction	6 (8.3)	100 (2.3)	0.008^†^
Epilepsy	0 (0)	45 (1.0)	1.000^†^
Chronic headache	1 (1.4)	131 (3.1)	0.726^†^

CVDs (cardiovascular diseases), SD (standard deviation), ECG (electrocardiogram), IQR (interquartile range), *p* (*p*-value).

^†^Fisher’s Exact test; ^‡^Independent *t*-test.

**Figure 2:**
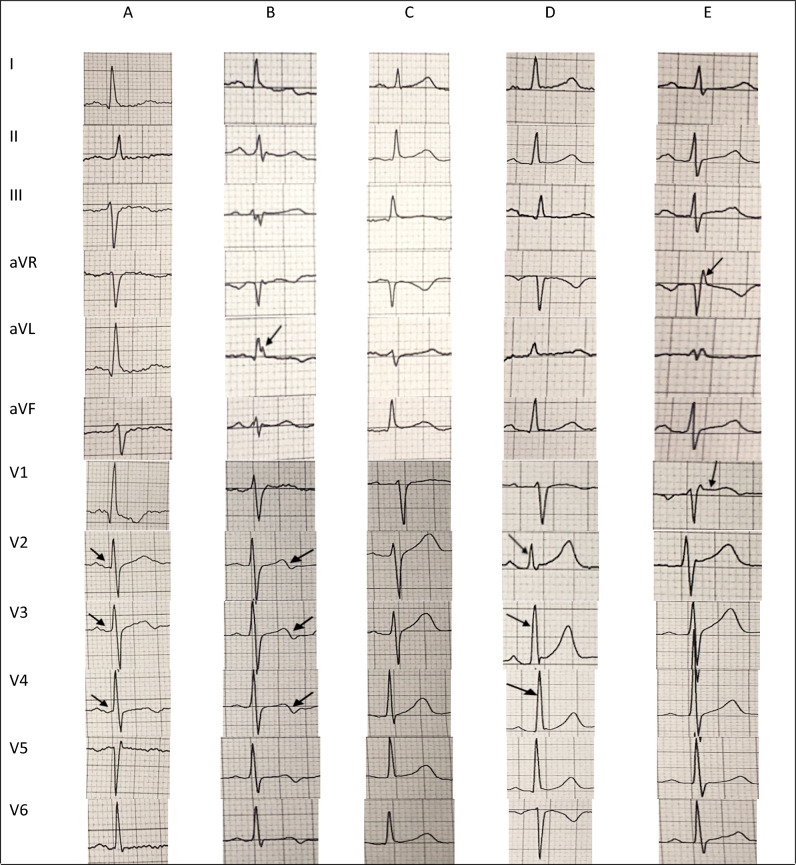
Baseline ECG characteristics of some the persons with short QT interval. (**A**) [case #1] a 52 Y/O male with a score 3 QTc, bradycardia (HR: 49) and AF. PQ segment depression is noted; (**B**) [case #2] a 43 Y/O male with a score 3 QTc. A notch in aVL lead in favor of scarring and a biphasic T wave are noted; (**C**) [case #17] a 46 Y/O male with a score 1 QTc, bradycardia (HR: 50), low voltage ECG and syncope; (**D**) [case #59] a 53 Y/O male with a score 1 QTc, relative bradycardia (HR: 59), AF, and counter clockwise rotation who expired due to SCD during the cohort; (**E**) [case #64] a 54 Y/O male with a score 1 QTc and relative bradycardia who was suspicious to the type II Brugada syndrome (a saddleback ST segment elevation in lead V1 and a prominent R wave in lead aVR, but not fulfilled R wave sign). Brugada test was not performed.

Table 3 depicts detailed data of the 72 subjects with varying degrees of SQTS probability. We could identify 2 subjects with high-probability SQTS (subject number 1 & 4) and 3 with intermediate-probability SQTS (subject number 2, 3 and 59). Subject #1 was a 52-year-old male with QTc of 318 ms and atrial fibrillation (AF). Subject #2 was a 43-year-old male with QTc of 318 ms. Subject #3 was a 58-year-old female with QTc of 324 ms with a positive history of palpitation (not scored). Subject #4 was a 48-year-old female with QTc of 317 ms and positive history of palpitation (not scored), sudden cardiac death (SCD) in her 18-year-old sister and another sister during infancy. And, subject #59: a 53-year-old male with QTc of 369 ms who expired due to SCD 2 years after performing ECG (Table 3, Fig. 2).

**Table 3:** Detailed data of subjects with QTc of lower than 370 ms at the baseline ECG.

SQTS susceptible									1st and 2nd degree relatives	
#	Sex	Age	QTc	ECG Score	Other ECG findings	AF	Hx of SCD	Syncope	Expire due to cardiac arrest^[ECA]^ or pacemaker implantation^[P]^	Infantile death
1^†^	M	52	318	3	B	+	−	−	−	−
2^‡^	M	43	318	3	Probable scar	−	−	−	−	−
3^‡^	F	58	324	3	LBBB	P	−	−	Mother and uncle^[ECA]^	−
4^†^	F	48	327	3	Low voltage	P	−	−	SCD in 18^Y/O^ sister, aunt^[P]^	Sister
5	M	40	339	2	B	−	−	−	−	−
6	M	60	341	2	−	+	−	−	−	−
7	M	40	348	2	B	−	−	−	−	−
8	M	47	349	2	Incomplete RBBB, B, ER	−	−	−	−	−
9	M	52	350	1	Low voltage	−	−	−	−	−
10	F	58	352	1	−	−	−	−	−	−
11	M	48	355	1	B	−	−	−	−	−
12	M	56	355	1	ER, B	−	−	−	−	Niece
13	M	66	355	1	−	+	−, revascularization	−	Father^[ECA]^	−
14	M	61	355	1	−	−	−	−	−	−
15	M	49	356	1	B	−	−	−	−	−
16	M	55	356	1	B, ER	−	−	−	−	Son, daughter
17	M	46	357	1	B, Low voltage	−	−	+	−	−
18	F	70	357	1	−	P	−, revascularization	−	−	−
19	M	61	358	1	B, ER	−	−	−	−	−
20	M	46	359	1	B	−	−	−	−	−
21	F	40	359	1	−	+	−	−	−	Twin children, 4 nephews
22	F	65	360	1	Low voltage	−	−	−	−	Son, daughter
23	M	51	360	1	−	−	−	−	−	−
24	M	59	361	1	B	−	−	−	−	−
25	F	42	361	1	Low voltage	P	−	−	−	−
26	M	41	362	1	−	−	−	−	−	−
27	M	41	363	1	−	−	−	−	−	−
28	M	46	363	1	B, ER	−	−	−	−	−
29	M	62	363	1	−	−	−	+	−	−
30	M	45	363	1	−	−	−	−	−	−
31	M	53	363	1	B	−	−	−	−	−
32	M	47	363	1	−	−	−	+	−	−
33	M	45	363	1	−	−	−	−	−	−
34	M	72	363	1	−	−	−	−	-	−
35	M	42	364	1	−	−	−	−	−	−
36	F	45	365	1	B, low voltage	−	−	−	−	−
37	M	47	365	1	B	−	−	−	−	−
38	M	48	365	1	−	−	−	−	Aunt^[ECA]^	−
39	M	68	365	1	−	−	−	−	−	−
40	M	60	365	1	−	−	−	−	−	−
41	M	54	366	1	−	−	−	−	−	−
42	M	46	366	1	−	−	−	−	Father^[ECA]^	−
43	M	62	367	1	B	−	−	−	−	−
44	F	41	367	1	−	−	−	−	−	−
45	M	49	367	1	−	−	−	−	Uncle^[P]^	−
46	M	58	367	1	−	−	−	−	Aunt^[ECA]^	−
47	F	50	367	1	LVH	P	−	−	Niece^[P]^	−
48	M	67	367	1	−	−	−	−	Aunt^[ECA]^	−
49	F	45	367	1	−	−	−	−	Father^[ECA]^	Brother
50	M	43	367	1	−	−	−	−	Uncle^[P]^	−
51	M	52	368	1	−	−	−	−	−	−
52	M	51	368	1	B	−	−	−	−	−
53	F	50	368	1	−	+	−	−	−	−
54	F	57	368	1	−	+	−	−	−	−
55	M	46	368	1	−	+	−	−	−	−
56	M	43	369	1	B	−	−	−	−	Niece
57	M	49	369	1	Low voltage, ER	−	−	−	−	−
58	M	43	369	1	−	−	−	−	−	−
59^‡^	M	53	369	1	B	+	+ (exp.), revascularization	−	Sister^[P]^	−
60	M	43	369	1	−	+	−, revascularization	−	Father^[ECA]^, mother^[P]^	−
61	M	43	369	1	−	−	−	−	−	−
62	F	44	369	1	−	−	−	−	Mother^[ECA]^, brother^[P]^	Sister
63	M	43	369	1	−	−	−	−	−	−
64	M	54	368	1	Probable BrS, B	+	−	−	−	−
65	M	53	368	1	B, ER	+	−, revascularization	−	−	−
66	M	66	369	1	B	−	−	−	−	−
67	M	64	369	1	Low voltage, B, ER	−	−	−	−	−
68	M	45	369	1	−	−	−	−	−	−
69	M	53	369	1	−	−	−	−	−	−
70	M	53	368	1	−	−	−	−	−	−
71	M	45	369	1	−	−	−	−	Aunt^[ECA]^	−
72	F	48	368	1	MI	+	−, revascularization	−	Father, mother and uncle^[ECA]^	−

*M* male, *F* Female, *B* bradycardia, *BrS* Brugada syndrome, *ER* early repolarization, *LBBB* left bundle branch block, *LVH* left ventricular hypertrophy, *exp.* expired, *Sx* syndrome, *MI* myocardial infarction, *P* palpitation, *SCD* sudden cardiac death, *Hx* history, *ECG* electrocardiogram, *CABG* coronary artery bypass graft, ^[ECA]^ (expired due to cardiac arrest), ^[P]^ (pacemaker implantation), Y/O (years old), msec (millisecond).

^†^High probability SQTS, ^‡^Intermediate probability SQTS.

**Figure 3:**
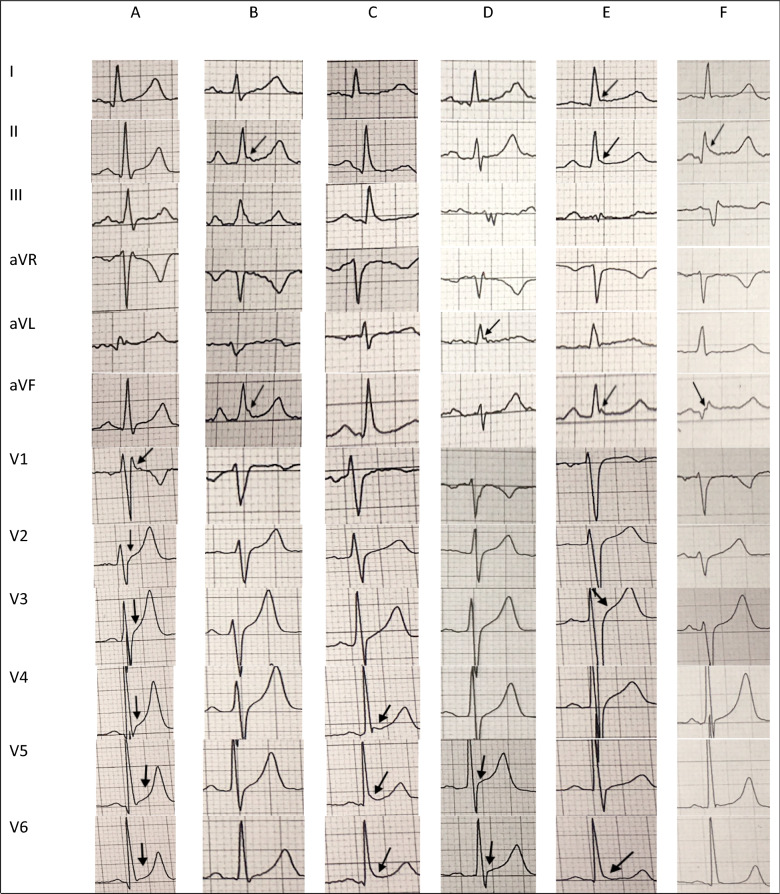
Baseline ECG characteristics of persons with short QT interval and early repolarization. (**A**) [case #8] a 47 Y/O male with a score 2 QTc, bradycardia (HR: 47), incomplete RBBB (rsr´ pattern in lead V1) and generalized ER; (**B**) [case #12] a 56 Y/O male with a score 1 QTc, a history of SCD in his niece and inferior leads ER; (**C**) [case #28] a 46 Y/O male with a score 1 QTc, bradycardia (HR: 51) and lateral leads ER; (**D**) [case #57] a 49 Y/O male with a score 1 QTc, low voltage ECG and lateral leads ER; (**E**) [case #65] a 53 Y/O male with a score 1 QTc, bradycardia (HR: 44) and inferolateral ER; (**F**) [case #67] a 64 Y/O male with a score 1 QTc, bradycardia (HR: 42), low voltage ECG and inferior lead ER.

Twenty (27.78%) subjects in SQTS-susceptible cohort showed SQTS-associated cardiac symptoms/events (symptomatic group). The frequency of AF and syncope were 16.67% and 4.17%, respectively. Moreover, bradycardia, early repolarization and low voltage were prevalent on their ECG; 33.33%, 11.11% and 11.11%, respectively. Female gender was significantly higher in symptomatic group compare to the asymptomatic group (*p* = 0.003). Other variables were not statistically different between the two groups (Table 4). Also, subject #64 was suspicious to have type II Brugada syndrome. His ECG showed a saddleback ST segment elevation in lead V1 and a prominent R wave in lead aVR, however its amplitude was not more than 0.3 mV- it was 0.2 mV- and did not fulfilled R wave sign (Figs. 2 and 3).

**Table 4:** Summary results of subjects with QTc of lower than 370 ms (SQTS susceptible group) at the baseline ECG.

	Total (n = 72)	Symptomatic (n = 20)^a^	Asymptomatic (n = 52)	*p*
**SQTS-associated cardiac symptoms/events during the cohort**				
Atrial fibrillation [frequency (%)]	12 (16.67)	–	–	–
Palpitation [frequency (%)]	5 (6.94)	–	–	–
Sudden cardiac death [frequency (%)]	1 (1.39)	–	–	–
History of syncope [frequency (%)]	3 (4.17)	–	–	–
**Baseline variables**				
Age, *year* (mean ± SD)	51.56 ± 8.41	52.25 ± 7.99	50.83 ± 8.41	0.517^‡^
Male/female ratio	1/0.26	1/0.82	1/0.13	0.003^†^
CVDs or MI [frequency (%)]	17 (23.61)	6 (30.00)	11 (21.15)	0.537^†^
**Past medical history**				
CVDs [frequency (%)]	11 (15.28)	4 (20.00)	7 (13.46)	0.485^†^
Myocardial infarction [frequency (%)]	6 (8.33)	2 (10.00)	4 (7.69)	0.667^†^
Chronic headache [frequency (%)]	1 (1.39)	1 (5.00)	0 (0)	0.278^†^
**Baseline ECG variables**				
QTc, ms (mean ± SD)	360.72 ± 11.72	356.90 ± 16.18	362.00 ± 9.11	0.096^‡^
Early repolarization [frequency (%)]	8 (11.11)	1 (5.00)	7 (13.46)	0.429^†^
Low voltage [frequency (%)]	8 (11.11)	3 (15.00)	5 (9.62)	0.677^†^
Bradycardia [frequency (%)]	24 (33.33)	5 (25.00)	19 (36.54)	0.414^†^
Cumulative ECG findings [frequency (%)]^b^	32 (44.4)	9 (45.00)	23 (44.23)	1.000^†^
**ECG score of SQTS probability [frequency (%)]**				0.095^¥^
1 (QTc < 370 ms)	64 (88.88)	16 (80.00)	48 (92.31)	
2 (QTc < 350 ms)	4 (5.56)	1 (5.00)	3 (5.77)	
3 (QTc < 330 ms)	4 (5.56)	3 (15.00)	1 (1.92)	
**1st and 2nd degree relatives (n = unknown)**				
Cardiac arrest or pacemaker implantation [frequency (%)]	16 (22.22)	7 (35.00)	9 (17.31)	0.123^†^
Infantile death [frequency (%)]	8 (11.11)	2 (10.00)	6 (11.54)	1.000^†^

*Hx* history, *CVDs* cardiovascular diseases, *MI* myocardial infarction, *SD* standard deviation, *ECG* electrocardiogram, *msec* millisecond, *p p*-value.

^a^Symptomatic SQTS was defined as being positive for any of the SQTS-associated cardiac symptoms/events.

^b^Cumulative ECG findings was defined as sum of the subjects in SQTS-susceptible cohort who had at least of the ECG findings, including early repolarization, low voltage, bradycardia, bundle branch, other conductive abnormalities.

^†^Fisher’s Exact test; ^¥^Pearson’s Chi square test; ^‡^Independent *t*-test.

Furthermore, a noticeable proportion- equal to 11.11%-reported the infantile SCD in their first- and second-degree relatives, which might represent the importance of genetic and pedigree patterns studies; for example, subject #16 was a 55-year-old male with QTc of 356 ms and positive history of infantile SCD in his son and daughter; Subject #21 was a 40-year-old female with QTc of 359 ms and positive history of infantile SCD in her twin children and 4 nephews; And, subject #22 was a 65-year-old female with QTc of 360 ms and positive history of infantile SCD in her son and daughter.

Finally, considering European Society of Cardiology criteria, we found at-least eight subjects with SQTS (subject number 1–5, 12, 16 & 21), which yielded an underestimated crude prevalence of 0.18%.

## Discussion

We presented the clinical profile and 4-year follow-up of 72 adult (≥ 40 years) subjects with short QT interval in one of the southern branches of the PERSIAN cohort. Findings were as following: (1) the prevalence of short QT interval in our normal adult population was 1.65% (n = 72, SQTS-susceptible group) with varying degrees of SQTS probability, which was within the range obtained by Japanese^14^, U.S.^13^ and Finnish^16^ cohorts (0.37 to 2.88% at 360 ms cut-off). Also, 4 subjects had QTc < 330 ms; (2) up to 27.78% of subjects in SQTS-susceptible group had symptoms, including AF (16.67%), palpitations (6.94%), unexplained syncope (4.17%), and SCD (1.39%), respectively; (3) at least, 2 subjects with high-probability SQTS and 3 with intermediate-probability SQTS identified according to Gollob’s criteria; (4) based on the European Society of Cardiology criteria, an underestimated crude prevalence of 0.18% was calculated for SQTS in our cohort; (5) at the baseline, SQTS susceptible group had a significantly higher myocardial infarction events; (6) bradycardia and early repolarization recognized in ECGs of 33.33% and 11.11% of SQTS-susceptible group; (7) SQTS-susceptible group had a significantly lower mean heart rate; (8) infantile SCD was found in 11.11% of the first- and second-degree relatives of SQTS-susceptible group, reflected that family screening for SQTS must carry out to avoid SCDs in mostly-asymptomatic subjects; (9) A significant male predominance observed in SQTS-susceptible group; (10) one suspicious case of Brugada syndrome was identified; (11) only gender was significantly different in symptomatic group of SQTS-susceptible cohort compare to the asymptomatic group.

Similar to in-line studies^14,16^, we found a male predominance in short QT interval group, supporting that QT interval is generally longer in females^21–23^. Although we could not assess this male predominance for SQTS, several studies have shown that the same is true for SQTS, which support the role of sex-specific parameters like sex hormones (especially testosterone level during puberty) modulation of potassium currents, genes located on the X chromosome, membrane ion channel availability, and intracellular signal transduction in pathogenesis of SQTS^10,14,24–26^.

In a recently published pooled analysis on 145 SQTS patients, 42.76% of patients were symptomatic (SCD: 33.79%, syncope: 21.38%, palpitation: 15.17%, and AF: 13.79%)^27^. We found that more than approximately one quarter of subjects with short QT interval had symptoms, comprising AF, palpitation, unexplained syncope, and SCD, respectively. In Miyamoto et al.^28^ cohort, the rate of AF was lower than our study (9.1% vs. 16.67%). Obviously, this is significantly higher than the 2.8% reported for adults in Iran^29^ and 2% in the general population in the world^30^, showing the presence of the same mechanism of an accelerated repolarization, shorter refractory period, and decreased action potential in the atrium. Several reports on childhood slow rate AF diagnosed with SQTS support this^31–34^.

Early repolarization usually accounts for as a benign ECG finding in normal population with a heterogenous prevalence, range 1–13%^35–41^. However, early repolarization might be associated with an increased risk of arrhythmic events like idiopathic ventricular fibrillation and the short QT interval^38,41^. That is, an uneven increase of early repolarization with a significant male precedence has been reported in short QT interval cases (6.1–30%)^14,40,41^ and SQTS patients (65%)^41^; nevertheless, this finding was reached up to 11.11% in SQTS-susceptible group of our study, predominantly involving inferolateral leads that was in agreement with previous reports^31,41^. The proposed common underlying mechanism for early repolarization is a reduction in inward repolarizing currents by loss of function mutations and/or an increase in outward repolarizing currents by gain-of-function mutations. It decreases the action potential duration (short QT interval), and increases the risk of reentrant mechanisms that can lead to AF and VF^28,42,43^. Noticeably, early repolarization is found in both short QT interval patients with mutations^44^ and without mutations in the known genes^43^, reflecting a variable genetic background for the association between short QT interval and early repolarization. By and large, the role of early repolarization, particularly inferior/inferolateral early repolarization, in risk stratification of cardiac events in individuals with short QT interval or SQTS is still unanswered, and studies on the association between long-term outcomes and presence of early repolarization in different QT interval groups are warranted.

The overlapping syndromes, concomitant Brugada-like (atypical) and SQTS, in a single patient with the same mutation and positive family history of SCD are reported in less reported variants including voltage-dependent calcium channel subunits (CACNA2D1, CACNA1C, CACNB2)^43^ and sodium channel protein (SCN5A)^45^. We identified a suspicious symptomatic (AF) case of Brugada-like ECG with short QT interval. This less understood varying genotype–phenotype relationship is believed to modulate by external factors (such as medication, fever, or electrolyte disorder)^46^ and genetic modifiers (such as ethnicity)^47^.

Another finding was bradycardia in one third of SQTS-susceptible group as well as a lower mean heart rate. One explanation might be the indecisive performance of correction formula. Whatsoever, some common physiologic effects i.e., ion channel gain or loss-of-function, hormonal effects or autonomic nervous system alterations (co-existence of lower heart rate and lower blood pressure in Anttonen et al.^16^, study) may incorporate in short QT interval, repolarization and attenuated sinus node activity.

In our cohort, infantile SCD was found in 11.11% of the first- and second-degree relatives of short QT interval group. Giustetto et al.^11^, showered that approximately half of SQTS patients have history of familial SCDs. In addition, SCDs in a close relative diagnose late in half of the families^48^. Furthermore, while most of cases never experience symptoms, SQTS can be highly malignant with a highest mortality rate preceding productive age^5^. As a result, SQTS represents as a “self-extinguishing” and “neglected” disease; that is, first- and second-degree relatives of an index patient must be screened and clinically assessed to avoid SCDs^7,49^.

Our study has at least four major limitations: (1) The calculated number of SQTS patients was a case for underestimation in our study. First, our cohort did not include subjects younger than 40 years, which rise the chance of underestimation. Previous studies have shown that SQTS might be more prevalent at the two age extremes, partially explained by two distinct underlying mechanisms^14,15^. Furthermore, the arrhythmogenic and malignant forms of SQTS are more likely to cause infantile SCDs and SCDs during 20 to 40 years of age^48^, and it appears that a short QT interval in a normal middle-aged subject may be considered as a benign condition^16^. This fact might be linked to the low rate of SCDs and symptomatic patients as well as lack of cases with extremely short QT interval (< 300 ms) and documented ventricular tachyarrhythmias in our cohort. Albeit, it should be noticed that in a SQTS cohort SCD did not occur in any of the patients during follow-up^50^. Second, Although Gollob’s diagnostic criteria relies on four items, we could not evaluate subjects for two items of both scoring criteria (items 3 and 4); (2) Due to the limited resources, we could not perform gene study and assess SQTS among first degree relatives of intermediate to high probability SQTS cases and symptomatic subjects with short QT interval. This could be a potential source of bias because these scenarios increase the chance of identifying more cases for such a disease that shows a robust familial clustering and most of cases diagnose incidentally, till being symptomatic or developing life-threatening events; (3) Although QT interval is gender-dependent, lower limit of QT interval is not described for males and females, separately. Hence, using a single cut-off value might exert bias in case finding, as this is expected to be longer in females than males. We partially dealt with this issue by using a higher cutoff value (370 ms). In addition, owing to the nature of our cohort- a normal adults’ population- we applied Bazett correction formula, which is shown to yield a consistent result at the range of heart rates encountered in healthy adults at rest^51^; (4) We could not assess the common causes of acquired short QT interval. Specifically, we were not able to assess clinical factors, including electrolyte abnormalities, that may have contributed to short QT intervals within our baseline measurements.

## Conclusion

In summary, the prevalence of short QT interval in our cohort was in line with previous studies. Nevertheless, the incidence of cardiac symptoms/events, familial SCDs and ECG derived specific findings were high amongst SQTS-susceptible index persons. But, most of the studied variables could not predict the symptomatic subjects, which emphasizes gene studies and family screening. Furthermore, the seriousness of short QT interval as well as contribution of clinical and paraclinical variables in risk stratification is still an open question. We believe that a long-term follow-up of young population within an analytical cohort study will be elucidative.

## Data Availability

The data underlying this article were provided by PERSIAN cohort (https://persiancohort.com/) by permission. Data will be shared on request to the corresponding author with permission of third party.
